# A Five-Year Study on Infestation and Abundance of Bat Flies (Hippoboscoidea: Streblidae) Under Severe Dry Season Conditions in the Tropical Dry Forest of Yucatan, Mexico

**DOI:** 10.1007/s13744-024-01130-z

**Published:** 2024-03-26

**Authors:** Ana Celia Montes de Oca-Aguilar, Martha Pilar Ibarra-López, Carlos N. Ibarra-Cerdeña

**Affiliations:** 1 Lab de Inmunología, Centro de Investigaciones Regionales “Dr. Hideyo Noguchi”, Univ Autónoma de Yucatán, Mérida, Mexico; 2 Dept de Ecología Humana, Centro de Investigación y de Estudios Avanzados del Instituto Politécnico Nacional (Cinvestav), Unidad Mérida, Mérida, Mexico; 3 Lab de Zoología, Dept de Ecología y Recursos Naturales, Centro Universitario de La Costa Sur, Univ de Guadalajara, Guadalajara, Mexico

**Keywords:** Neotropical region, Ectoparasites, Dipteran, *Artibeus jamaicensis* Leach, *Pteronotus parnellii* Gray, Environmental variables

## Abstract

In Mexico, few studies have explored how environmental conditions in tropical dry forests (TDF) influence bat fly load even though, according to climate change scenarios, this ecosystem will experience a drier and warmer climate. Such an extension of the dry season in these ecosystems could have dramatic consequences for biodiversity, particularly in regions with plains where animals do not have elevational climate shifts. The present study therefore evaluates the effect of prevailing environmental conditions during 2015–2019, as well as host body conditions, on the infestation and abundance of bat-specific ectoparasites and the composition and bat fly load in the dry season of a TDF in Yucatan. Since Yucatan has an essentially flat and low-lying topography, organisms cannot escape from the predicted extreme conditions with elevational shifts. This region is therefore an excellent location for assessment of the potential effects of warming. We collected 270 bat flies from 12 species. Three streblid species (*Nycterophilia parnelli* Wenzel, *Trichobius johnsonae* Wenzel, and *Trichobius sparsus* Kessel) are new records for Yucatan. Our overview of the dry season bat ectoparasite loads reveals low values of richness and prevalence, but high aggregation. Our models detected significant differences in ectoparasite infestation and abundance over the years, but the environmental and body host condition variables were unrelated to these. We report that pregnant females are parasitized to a greater extent by bat flies during the dry season, which generally represents the season of most significant nutritional stress.

**Supplementary Information:**

The online version contains supplementary material available at 10.1007/s13744-024-01130-z.

## Introduction

Tropical dry forest (TDF) is recognized worldwide for its remarkable biodiversity and endemism (Pennington et al. 2009), yet it is a severely threatened ecosystem (Miles et al. 2006). TDF is markedly seasonal, driving the faunal assemblage due to dynamic and structural variation of the available resources. The rainy season is very wet (with records of 1 to 3 m of rainfall) (Janzen 1988) while in the dry season (which lasts 3 to 8 months per year), the average annual rainfall is less than 100 mm, giving rise to predominantly deciduous forest vegetation and a complex mosaic of different habitat types available for the inhabiting species (Van Bloem et al. 2004). Consequently, during the dry season, some animals migrate to humid refuges (Janzen 1988) while for other species, the climate presents a physiological (e.g., nutritional) challenge (Lewanzik et al. 2012; Martínez-Mota et al. 2016; Orkin et al. 2019). Severe conditions that persist in the dry season seem to influence the fitness of vertebrates (de Araujo et al. 2017) or the biological interactions that exert selective pressures on animals, such as host-parasite relationships. Parasites regulate host populations by impacting their fitness in several non-exclusive ways due to increased energy demand (due to blood consumption and infections). Individuals with high loads of ectoparasites will therefore experience lower growth rates, causing a reduction in body mass and lowering reproductive success (Lehmann 1993; Krasnov et al. 2006). As such, the effects of ectoparasites must be synergic in stressful environments, such as those with high temperatures and changes in precipitation patterns (Dawson et al. 2005; Altizer et al. 2006; Scharsack et al. 2016; Musgrave et al. 2019). Current climate trends show that precipitation patterns in the TDF will change, with an overall reduction in rainfall and intensification of dry intervals or longer dry seasons (Stan and Sanchez-Azofeifa 2019). These changes could lead to physiological stress (Welbergen et al. 2008), affecting the interaction and dynamics of parasites and hosts (Barrett et al. 2013; Morley and Lewis 2014). However, the climatic crisis is not the only factor that endangers the wildlife, since human land use is responsible for steadily reducing the forest cover (Ureta et al. 2022). The Yucatan Peninsula is mainly a plain region with a flat topography (Vázquez-Dominguez and Arita 2010) and it is therefore impossible to use altitudinal shifts to find new refuges (Colwell et al. 2008).

Bats are one of the most important functional groups of the tropical ecosystems, yet our understanding of bat-parasite interactions is still incipient. Diptera species of the family Streblidae (superfamily Hippoboscoidea) are one of the most diverse, abundant, adapted, and specialized ectoparasites that feed on bat blood (Wenzel et al. 1966; Dick and Patterson 2006, 2007). Of the 245 species recorded worldwide, 161 have been documented in America (Dick and Graciolli 2018). Most Streblid species are members of Phyllostomidae (Dick and Patterson 2006), one of the most diverse families of bats. Streblid bat flies are grouped into three subfamilies: Nycterophilinae, Trichobinae, and Streblinae (Dick and Graciolli 2018). Although the life cycle of bat flies occurs entirely on the host, the pupae are deposited in the bat roost and, following pupation, the emerged adult must rapidly find a bat host (Dick and Patterson 2006). The bat-streblid relationship tends to be specific, so a strong association between local flies and bat species richness would be expected (Wenzel et al. 1966; Wenzel 1976; Barbier and Bernard 2017).

Two-level general factors influence bat fly species richness, abundance, intensity, and prevalence. On the one hand, host accessibility is influenced by habitat and environmental conditions affect the habitat structure and the dynamic of the bat roost (Ter Hofstede and Fenton 2005; Patterson et al. 2007; Bolívar-Cimé et al. 2018; Vieira et al. 2019). On the other hand, the host quality is driven by the host age, sex, reproductive condition, health status (Rui and Graciolli 2005; Patterson et al. 2008), and bat behavior and size (Marshall 1982). However, few studies have focused on analyzing the influence of environmental conditions, such as temperature and precipitation, on bat flies. While some studies suggest a lack of association between the quantity of precipitation and the abundance of ectoparasites (Barbier et al. 2019), others indicate a strong influence of climate conditions or environmental differences on species composition and bat fly load (Pilosof et al. 2012; Patrício et al. 2016; Zarazúa-Carbajal et al. 2016; Eriksson et al. 2020; Colín-Martínez et al. 2018). Some studies have shown that seasonality and microclimatic variations in temperature generate a disparity of response in terms of parasite load parameters in different host species (Pilosof et al. 2012; Tlapaya-Romero et al. 2021).

In Mexico, the order Chiroptera comprises 137 species of 67 genera and eight families (Ramírez-Pulido et al. 2014). However, only 69 species of bat flies from 17 genera have been recorded (Trujillo-Pahua and Ibáñez-Bernal 2020; Tlapaya-Romero et al. 2023), many of which are only recently discovered species. Although ecological studies of bat flies and host interactions are scarce, most were conducted in the tropical dry forest from the states of Puebla, Oaxaca, and Jalisco (Rivera-García et al. 2017; Salinas-Ramos et al. 2018; Zarazúa-Carbajal et al. 2016; Hernández-Martínez et al. 2019; Tlapaya-Romero et al. 2021).

In the Yucatan Peninsula biogeographical province, Chiroptera is the most diverse order of mammals, with 64 species. The family Phyllostomidae is the most diverse in this order (Sosa-Escalante et al. 2013). In this province, studies of bat flies have focused on increasing taxonomic knowledge (Pearse and Kellogg 1938; Hoffmann 1953; Wenzel 1970; Peterson and Hurka 1974; Guerrero and Morales-Malacara 1996; Lira-Olguin et al. 2021), with descriptions of infection prevalence in bat communities (Cuxim-Koyoc et al. 2015). Few studies have focused on describing parasite load at the individual host or habitat level (Bolívar-Cimé et al. 2018). No studies have been published addressing temporal variation patterns in bat fly loads associated with climatic conditions. However, this is important since the evidence suggests that climate change in Mexico has been occurring unequally among the different biogeographical provinces that group or concentrate the biological diversity of that country (Cuervo-Robayo et al. 2020). It has been suggested that, in the last two decades, the Yucatan Peninsula (and other neotropical regions) has exhibited a more pronounced decrease in rainfall (Cuervo-Robayo *Op. cit*), with a clear and constant warming trend (0.001 °C/year, Andrade-Velázquez et al. 2021). This is most evident in Yucatan state, where TDF is dominant (Prieto-Torres et al. 2016).

In the present study, we characterized the infra-community of the bat fly host over 5 years (2015–2019) during the dry season, which represents the most significant water stress period in the TDF of Yucatan. We focused on bat composition, sex, and physical status (size, height, weight) to evaluate inter-annual change in ectoparasite loads in bat species of highest abundance (*Artibeus jamaicensis* Leach -Phyllostomidae- and *Pteronotus parnellii* Gray -Noctilonoidea-), as well as determining whether bat fly loads are associated with biological (sex, physical status [size, height, weight]) and environmental conditions. We hypothesize that the magnitude of the variation in environmental conditions across the different dry periods will influence the host condition (i.e., host quality) and ectoparasite load relationship. We expect that, in years when environmental conditions are more severe (e.g., decreased precipitation, increased temperature, and solar radiation), the host condition will decrease, and this will be reflected in the ectoparasite load.

## Materials and methods

### Study area

**Fig. 1:**
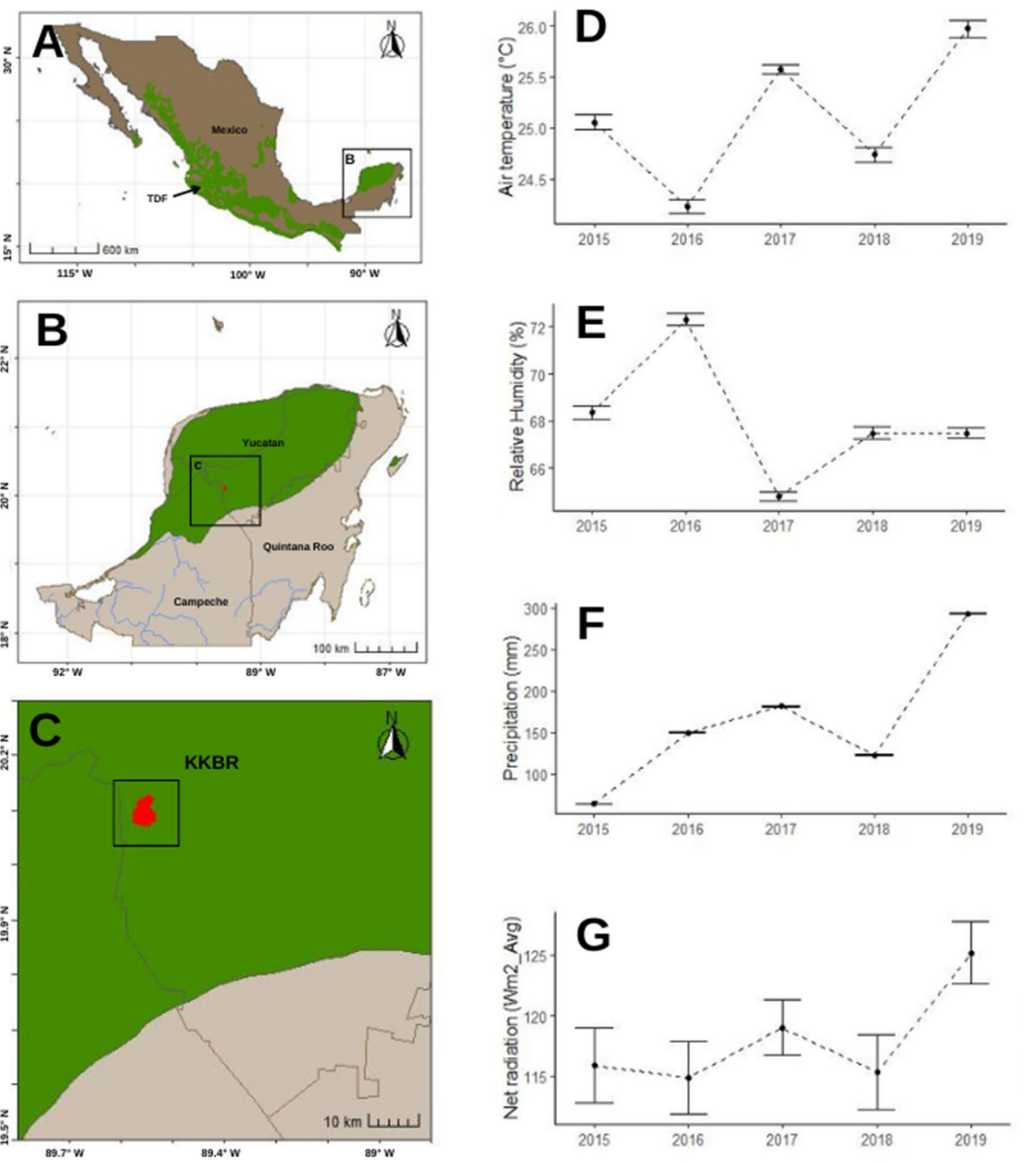
Geographic location of Kaxil Kiuic Biocultural Reserve and recording of environmental variations over 5 years. Distribution of tropical dry forest (TDF) in Mexico (**A**). Distribution of TDF on the Yucatan Peninsula (**B**). Kaxil Kuic Biocultural Reserve (KKBR) (**C**). Environmental variables (mean + standard error): air temperature (°C) (**D**), relative humidity (%) (**E**), precipitation (mm) (**F**), and net radiation (Wm^2^ avg) (**G**). Years with the same letter did not present significant differences (*p* < 0.05) according to the Dunn post hoc test

The study area is the Kaxil Kiuic Biocultural Reserve (KKBR), which presents a continuous TDF (Fig. 1). KKBR (20°5′20°7′N; 89°32′89°34′W) is part of the Puuc Biocultural State Reserve and has an area of 1642 ha of TDFs that have existed for more than 100 years after their ancient occupation by Mayan people (Essens and Hernández-Stefanoni 2013). The area constitutes an important biological corridor for TDFs that connects the medium-sized forests of the central Yucatan Peninsula with the wetlands of western Yucatan and Campeche. The trees of KKBR reach a height of 13–20 m, dominating the arboreal and shrubby elements with scarce climbing plants and epiphytes (Dupuy et al. 2012).

The climate is warm subhumid (Aw), with mean annual temperature and precipitation of 26 °C and 1000–1200 mm, respectively (Orellana et al. 2003). The rainy season lasts from June to October, with an annual precipitation of 763–916 mm. The dry season covers November to May, with an annual precipitation of 237–284 mm (Morffi-Mestre et al. 2020). Our fieldwork was conducted for five consecutive years (2014–2019) in the dry season. According to the annual precipitation records, a decrease was recorded during the first 3 years, with a slight increase in 2017 and 2018 (Morffi-Mestre et al. 2020).

### Environmental variation in the dry seasons

To describe and quantify the difference between environmental variables over the years at a local scale, we used environmental data from the weather station of KKRB. Data pertaining to temperature (Temp: average, minimum, maximum, °C), precipitation (PR, mm), air temperature (AT, °C), relative air humidity (RH, %), and net radiation (NR) were recorded by an automatic weather station every 30 min for the entire study period. We analyzed the correlation between variables to discard highly correlated variables (*p-value* < 0.001, *r* = 0.75). Finally, we used four of the original seven variables (AT, RH, PR, and NR) in subsequent analyses. To evaluate differences between the environmental variables among years, we performed a Kruskal–Wallis test and a post hoc test with Bonferroni correction using the dunn.test package in R v. 3.1.0. Our analysis revealed significant differences among years in AT (Kruskal–Wallis = 568.7441, *df* = 5, *p-value* = 0), RH (Kruskal–Wallis = 3049.5712, *df* = 5, *p-value* = 0), PR (*Kruskal–Wallis* = 118.6879, *df* = 5, *p-value* = 0), and NR (*Kruskal–Wallis* = 303.98, *df* = 5, *p-value* < 2.2e − 16) (Fig. 1 and Table S1). A decrease in NR was detected in 2015–2018, and an increase in precipitation and AT in 2019, but a decrease in RH was observed (Fig. 1). This variable was used in the subsequent analysis.

### Capture and identification of bats and flies

We captured bats in five consecutive years (2015–2019). Fieldwork was conducted for 22 intermittent days during March–May. We used around two and five mist nests per year (6, 9, and 12 m) that remained open for 4 h after sunset and were reviewed at 30-min intervals. These mist nets were established at 11 different sites in the forest including one site in front a cave. Throughout the study period, we sampled 22 nights (2151 m/net for 4 h), yielding a total sampling effort of 8604 m^2^ h (Straube and Bianconi 2002).

Taxonomic identification of the bats followed Medellín et al. (2008) and Álvarez-Castañeda et al. (2017). For each species of bat captured, the following biological data were recorded: sex, reproductive condition (inactive, post-lactating, lactating, pregnant, testicle position), weight (grams), total length, forearm length, tail length, and ear length. Ectoparasites were collected using forceps and preserved in 96% ethanol. Bat flies were observed under a VELAB Serie S © stereomicroscope for taxonomic determination using the keys and descriptions provided in Wenzel et al. (1966) and Wenzel (1976).

### Data analysis

We described ectoparasite load parameters at two levels, in the whole bat community and in terms of the associations with environmental factors and host conditions in the two most abundant bat species (the frugivore *A. jamaicensis* and in the insectivore *P. parnellii*). We described the bat fly infra-community according to the parasite load parameters previously defined by Bush et al. (1997) and calculated mean abundance (MA), mean intensity (MI), aggregation (D), and prevalence (P) for each host species registered in the study area. We calculated parasite aggregation (D), represented by the discrepancy index (Poulin 1993), which allowed us to infer how the intrapopulation of bat flies was distributed among their hosts. Values of this index range from 0 (no aggregation) to 1 (high aggregation) (Poulin 2007). We calculated these values and estimated their confidence intervals (95% CI) by bootstrapping with 2500 iterations (Rózsa et al. 2000). All parameters related to parasite load and significant differences were calculated using the Quantitative Parasitology (QPew) program, version 1.0.15.

We assessed differences in ectoparasite load parameters across years using Fisher’s exact test with P data and Bootstrap test with 2500 iterations with MA, MI, and D data (Reiczigel et al. 2019). Specifically, *A. jamaicensis* and *P. parnellii* were exclusively used for these and subsequent analyses due to their higher abundance (*n* > 30). We conducted a Spearman correlation analysis to explore the association of these parameters with environmental conditions (AT, NR, RH, PR).

Then, we performed a generalized linear model (GLM) to assess the effects of year, environmental conditions (AT, NR, RH, P), body size (mm), host sex, and BIC (bat body index condition: weight of host/forearm length) on infestation status (infected/no infected) and abundance. A GLM with a Binomial error structure was used for the infestation status and a Poisson error structure for the abundance data (all ectoparasite species together). Given the excessive number of zeros in our abundance data, a zero-inflated Poisson (ZIP) error structure GLM was conducted using the pscl package. We employed the Vuong test to compare ZIP with a GLM model with a significant *p-value* of ≤ 0.05 to select the best model according to Akaike’s Information Criterion (AIC). We also assessed the effect of host condition on infestation and ectoparasite abundance per sex (male: sexually inactive, age -adult/younger-, scrotal testes, abdominal testes, and inguinal testes; female: sexually inactive, age -adult/younger-, lactating and pregnant). For each predictor variable, we estimated the variance inflation factor (*VIF*) using the car package (Jou et al. 2014). Six numerical predictor variables presented lower *VIF* values (≤ 1.4), suggesting independence among them (Jou et al. 2014). In contrast, body size (*VIF* value 2.35) was removed from the models. Model selection procedures were based on AIC using the gmulti package. The best models were those that had lower *AIC* values.

## Results

### Specific richness and abundance of hosts

A total of 285 individual bats of 17 species were caught and sampled during the collection period. The 17 species were distributed among the families Phyllostomidae (*n* = 167), Mormopidae (*n* = 104), Vespertilionidae (*n* = 9), Molosidae (*n* = 4), and Natalidae (*n* = 1) (Table 1). *Artibeus jamaicensis* (*n* = 123), *P. parnellii* (*n* = 80), *Desmodus rotundus* Geoffroy (*n* = 21), and *Pteronotus davyi* Gray (*n* = 15) were the most abundant bat species. A total of 68 individuals of six species were parasitized by Streblidae (Table 1) while 12 bat species were not parasitized by flies (Table 1).

**Table 1:** Infra-communities of bat flies (Streblidae) collected in hosts from 2014 to 2019 in the Kaxil Kiuic Biocultural Reserve

Host species	*n*	Infested bat (%)	Bat fly species				P (95%CI)	MA (95% CI)	MI (95% CI)	D (95% CI)
				*n*	♀ *n*	♂ *n*				
Family Phyllostomindae										
*Artibeus jamaicensis* Leach	123	18 (15)	*Trichobius intermedius* Peterson and Hürka *Megistopoda aranea* (Coquillett)	23 1	18 -	5 1	0.15 (0.69–0.19) 0.008 (NA)	0.18 (0.09–0.36) 0.008 (0–0.02)	1.53 (1.07–2.76) 1 (NA)	0.91 (0.85–0.94) 0.98 (0.95–0.98)
*Artibeus lituratus* Olfers	5		-	-	-	-	-	-	-	-
*Dermanura phaeotis* (Miller)	8		-	-	-	-	-	-	-	-
*Dermanura tolteca* (Saussure)	1		-	-	-	-	-	-	-	-
*Dermanura watsoni* (Thomas)	1		-	-	-	-	-	-	-	-
*Desmodus rotundus* Geoffroy	21	6 (29)	*Trichobius parasiticus* Gervais *Strebla wiedemanni* Kolenati	17 4	8 3	9 1	0.27 (0.11–0.52) 0.14 (0.03–0.36)	0.76 (0.28–1.86) 0.19 (0–0.42)	2.67 (1.5–4.67) 1.33 (1–1.67)	0.78 (0.65–0.88) 0.84 (0.68–0.90)
*Glossophaga soricina* Pallas	6		-	-	-	-	-	-	-	-
*Sturnira parvidens* Goldman	3		-	-	-	-	-	-	-	-
Family Mormopidae			-	-	-	-	-	-	-	-
*Pteronotus parnellii* Gray	80	40 (50)	*Trichobius yunkeri* Wenzel *Trichobius intermedius* *Nycterophilia coxata* Ferris *Nycterophilia parnelli* Wenzel	123 2 62 33	63 2 22 14	60 - 40 19	0.41 (0.30–0.52) 0.012 (NA) 0.23 (0.15–0.34) 0.12 (0.062–0.22)	3.73 (2.85–4.91) 0.02 (0–0.07) 0.76 (0.43–1.31) 0.41 (0.16–0.75)	3.73 (2.88–4.97) 2 (NA) 3.26 (2.26–4.95) 3.3 (2–4.3)	0.75 (0.67–0.82) 0.98 (0.93–0.98) 0.85 (0.79–0.91) 0.90 (0.84–0.95)
*Pteronotus davyi* Gray	15	2 (13)	*Trichobius johnsonae* Wenzel *Trochobius sparsus* Kessel	1 1	- 1	1 -	0.067 (NA) 0.067 (NA)	0.06 (0–0.2) 0.06 (0–0.2)	1 (NA) 1 (NA)	0.87 (0.62– 0.87) 0.87 (0.62– 0.87)
*Mormoops megalophylla* (Peters)	9	2 (22)	Wenzel *Trichobius sparsus*	2 1	2 1	- -	0.111 (NA) 0.008 (NA)	0.11 (0–0.33) 0.22 (0–0.67)	1 (NA) 1 (NA)	0.80 (0.4–0.8) 0.80 (0.5–0.8)
Family Vespertilionidae										
*Eptesicus furinalis* D’Orbigny and Gervais	1		-	-	-	-	-	-	-	-
*Lasiurus ega* (Gervais)	3		-	-	-	-	-	-	-	-
*Myotis keaysi* Allen	3		-	-	-	-	-	-	-	-
*Rhogeessa aeneus* Goodwin	2		-	-	-	-	-	-	-	-
Family Moolosidae				-	-	-	-	-	-	-
*Molossus ater* Geoffroy	4		-	-	-	-	-	-	-	-
Family Natalidae										
*Natalus stramineus* Gray	1		-	-	-	-	-	-	-	-
Total	286	68 (24)								

*NA* insufficient data with which to calculate confidence intervals

*P* prevalence (%), *MA* mean abundance, *MI* mean intensity of infestation, *D* index of aggregation, *CI* confidence intervals

### Bat fly diversity and loads

**Fig. 2:**
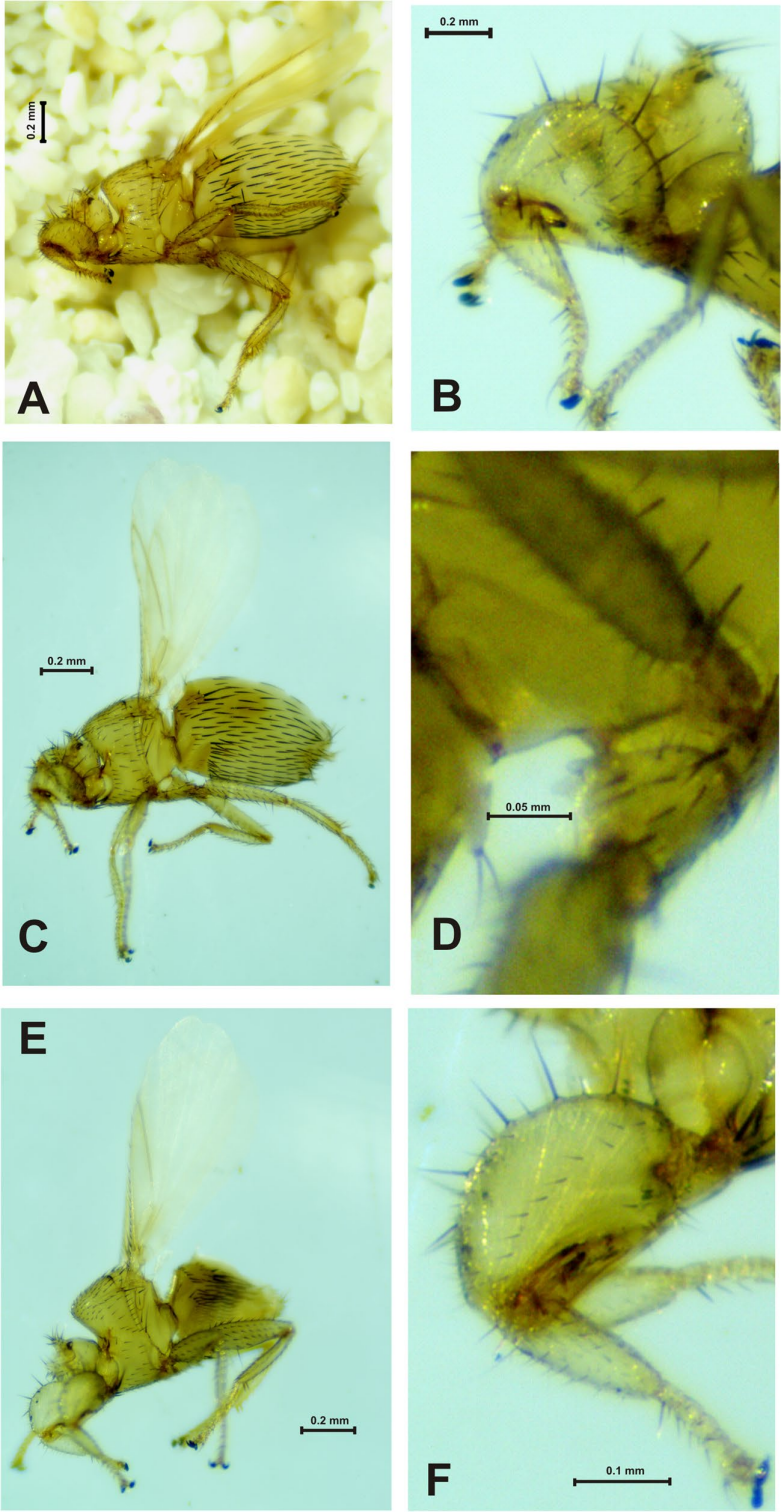
*Nycterophilia* species recorded in Kaxil Kiuic Biocultural Reserve. *Nycterophilia coxata* (♀), lateral view (**A**); profemur, lateral view (**B**). *Nycterophilia parnelli* (♀), lateral view (**C**); metacoxal spur (**D**). *Nycterophilia mormoopsis* (♀), lateral view (**E**); profemur, lateral view (**F**)

**Fig. 3:**
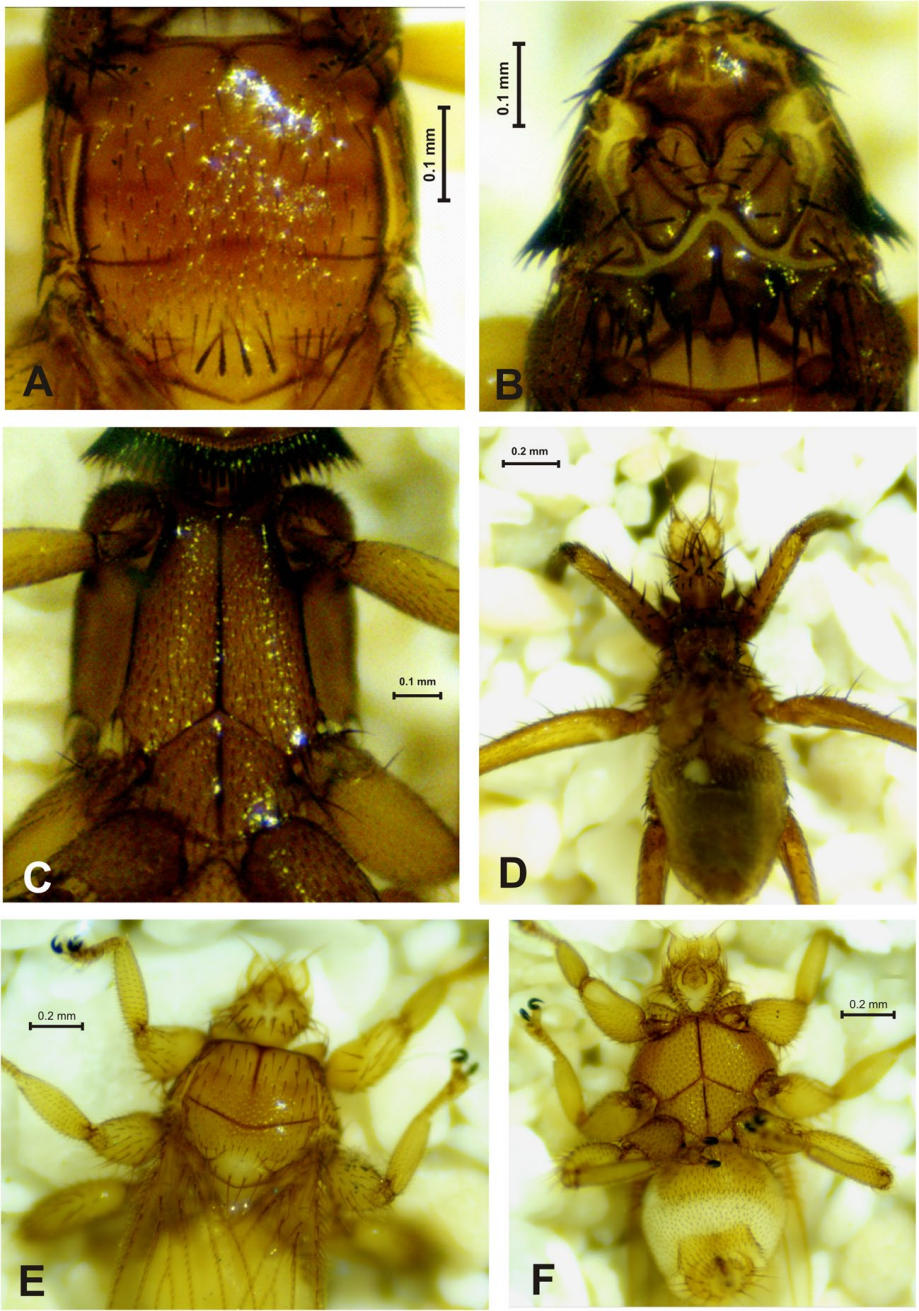
Streblidae species collected in Kaxil Kiuic Biocultural Reserve. *Strebla wiedemanni* (♀), dorsal view of the thorax (**A**), dorsal view of the head (**B**), and ventral view of the thorax (**C**). *Megistopoda araneae* (♂), dorsal view (**D**). *Trichobius intermedius* (♀), dorsal view (**E**) and ventral view (**F**)

We collected 270 bat flies of the family Streblidae, distributed across 10 species and four genera (Table 1). *Trichobius* Gervais (*n* = 5 species) was the genera with the highest number of species, followed by *Nycterophilia* Ferris (Fig. 2) (*n* = 3 species). *Strebla* Wiedemann and *Megistopoda* Macquart (Fig. 3) were presented by only one species each. The number of species per genus was consistent with the range of host-parasitized species.

**Fig. 4:**
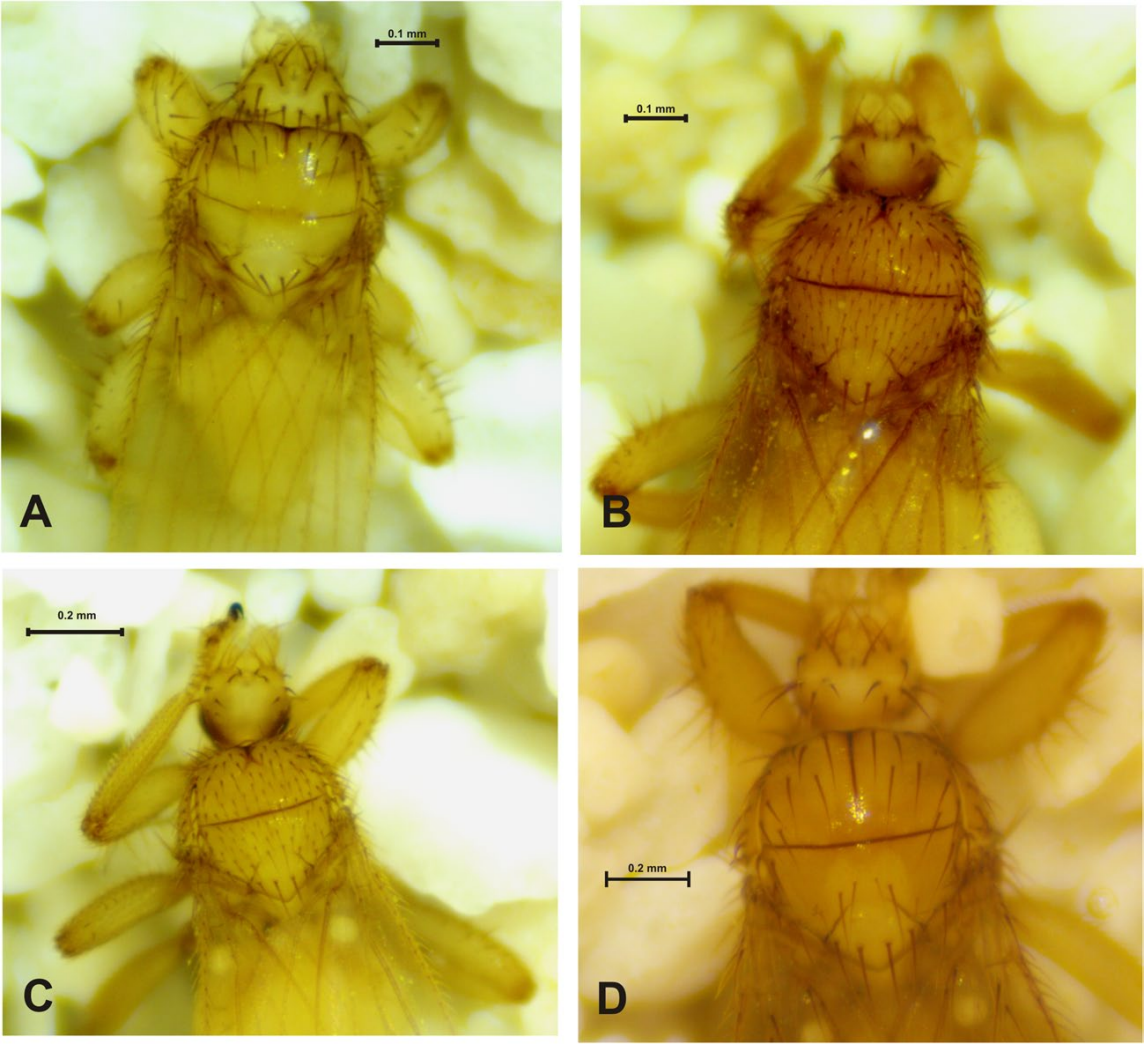
*Trichobius* species collected in Kaxil Kiuic Biocultural Reserve. *Trichobius parasiticus* (♀), dorsal view (**A**). *Trichobius yunkeri* (♂), dorsal view (**B**). *Trichobius johnsonae* (♂), dorsal view (**C**). *Trichobius sparsus* (♀), dorsal view (**D**)

The host with the highest bat fly species richness was *P. parnellii* (*n* = 3 species), while *A. jamaicensis*, *Desmodus rotundus, P*. *davyi*, and *Mormoops megalophylla* (Peters) were infested by two bat fly species (Table 1). Considering the association, 80% of the bat flies (10 species) were specific to only one bat species. *Trichobius intermedius* Peterson and Hurka (Fig. 2E, F), *T. parasiticus* Gervais (Fig. 4A), and *Strebla wiedemanni* Kolenati (Fig. 3A–C) were recorded on two bat species (Table 1). Likewise, only one specimen of *Megistopoda aranea* (Coquillett) (Fig. 3D) and one of *T. johnsonae* Wenzel (Fig. 4C) were collected on *A. jamaicensis* and *P. davyi*, respectively.

The host-ectoparasite relationship between *P. parnellii* and *Trichobious yunkeri* Wenzel had the highest prevalence (40.7%), while the second highest prevalence values were for *N. coxata* Ferris (23.5%) on *P. parnellii*, and *T. intermedius* (14.6%) on *A. jamaicensis.* The mean intensity of infestation and mean abundance was highest for *T. yunkeri* (MI = 3.73, MA = 3.73), *Nycterophilia coxata* Ferris (MI = 3.26, MA = 0.76), and *N. parnelli* Wenzel (MI = 3.3, MA = 0.41) on *P. parnellii* (Table 1). Mean intensity and mean abundance was also highest for *T. intermedius* (MI = 1.53, MA = 0.18) on *A. jamaicensis*. Among the most representative host species in the sample, the ectoparasitic flies with the highest aggregation values were *T. intermedius* (0.908) on *A. jamaicensis*. High aggregation values were also recorded for *N. parnellii* (0.903) and *N. coxata* (0.852) on *P. parnellii*.

### Bat fly load and its relationship to host attributes and environmental conditions

According to Fisher’s exact test, the prevalence of bat flies on *A. jamaicensis* and *P. parnellii* was higher in 2018 and 2019 (Table 2), with no significant differences between the sexes (Table S1). Significant differences in MA, MI, and D were observed between years in *P. parnelli*, whereas *A. jamaicensis*, influenced by its ectoparasite load, only exhibited differences in aggregation (Table 2). For *P. parnelli*, MA was higher in 2015, 2018, and 2019, while MI was higher in 2015 and 2019. Both bat species showed lower aggregation values in 2018, while *P. parnelli* also recorded low values in 2015. No correlation was found between any ectoparasite parameters and environmental variables (Table S2).

**Table 2:** Comparison results of Streblid bat fly load parameters collected on *Artibeus jamaicensis* and *Pteronotus parnellii* over 5 years in the dry seasons

Species	Parameter	2015 (a)	2016 (b)	2017 (c)	2018 (d)	2019 (e)
	*n*	6	18	18	18	21
*P. parnellii*	P (95% CI)	66.7 (0.094–0.992)	16.7 (30–41)	10 (1.3–33)	78.9 (54.4–93.4)	70.6 (44.0–89.7)
			d**, e**	d***, e***	b**, c***	b***, c***
	MA (95% CI)	5.33 (0–10.3)	0.83 (0.11–2.31)	0.15 (0–0.47)	3.42 (2.21–5)	5.82 (3.35–8.91)
			e**	d***, e***	c***	b**, c***
	MI (95% CI)	8 (1–8)	5 (2–7)	1.5 (1–1.5)	4.33 (3.07–5.8)	8.25 (5.58–11.7)
				d**, e***	c**	c***
	D (95% CI)	0.46 (0 – 0.5)	0.83 (0.657–0.895)	0.86 (0.7–0.9)	0.46 (0.36–0.67)	0.51 (0.358–0.683)
		b**, c**	a**	a**, d**	c**	
	n	33	14	37	10	30
*A. jamaicensis*	P (95% CI)	9.1 (1.9–24.3)	7.1 (0.2–33.9)	5.4 (0.7–18.2)	50 (18–81.3)	16 (5.6–34.7)
		d**, e**			a**	a**
	MA (95% CI)	0.30 (0.03–0.97)	0.0714(0–0.214)	0.08(0–0.27)	0.50(0.1–0.7)	0.16(0.033–0.3)
	MI (95% CI)	3.33 (1–5)	1 (NA)	1.5 (1–1.5)	1 (NA)	1(NA)
	D (95% CI)	0.91 (0.81–0.94)	0.87 (0.6– 0.867)	0.93 (0.842–0.947)	0.45(0.16–0.63)	0.806(0.58–0.87)
		d**		d**,	a**, c**	

The number of bats examined per species per year is specified (*n*). *P*, prevalence (%), *MA*, mean abundance; *MI*, mean intensity of infestation; *D*, index of aggregation; *CI*, confidence intervals. Statistical significance represent by letters (lower, *p* < 0.05**, 0.001***)

The results of the ZIP and GLM models for bat fly infestation status and abundance are summarized in Table 3. Our dataset on infestation aligns with the prevalence result dataset and the correlation analysis. For the infestation dataset, the best model was defined by species and year (*AIC* = 177.9, Table S3), with no significant interaction between these predictors. Infestations were higher in *P. parnellii* than on *A. jamaicensis*. Our model also detected significant differences between years, with high infestation values presented in 2018 (Table 3).

**Table 3:** Summary of ectoparasite infestation and abundance analysis results for independent variables

Component	Estimate	Std. Error	*z* value	Pr( >|z|)
A)				
Intercept	− 2.08	0.507	− 4.11	3.91E − 05***
Species (*P. parnellii*)	1.88	0.42	4.43	9.15E − 06***
Year: 2016	− 1.22	0.79	− 1.53	0.12
Year: 2017	− 1.46	0.76	− 1.90	0.05
Year: 2018	1.77	0.68	2.60	0.00918***
Year: 2019	0.82	0.60	1.36	0.17
B)				
Intercept	0.64	0.75	0.86	0.39
Species (*P. parnellii*)	1.61	0.47	3.41	0.0006***
Year: 2016	− 0.79	0.34	− 2.30	0.0212**
Year: 2017	− 1.81	0.69	− 2.61	0.0088***
Year: 2018	− 1.01	0.24	− 4.07	4.55E − 05***
Year: 2019	− 0.61	0.24	− 2.52	0.0115**
Host sex (M)	− 0.88	0.25	− 3.51	0.0004***
Host BCI	1.22	0.96	1.27	0.20

Generalized linear model results using a Poisson error structure for infestation data **(A)**, and zero-inflated Poisson (ZIP) error structure GLM results for abundance data **(B)**. Significant *p*-values are in bold font (0.05**, 0.001***). *BCI*, body condition index, *M*, male

For abundance data, the best model was defined by species, year, and sex (AIC = 551.72, Table S3). No interaction between predictors was considered significant. Bat fly abundance was higher in *P. parnellii* that in *A. jamaicensis*. The ZIP model detected significant differences between years and sex (Table 1). Ectoparasites were more abundant in females (80%) than in males (20%) (Table S4). Significantly lower abundance was observed in 2016 and 2017, while higher abundance was recorded in 2018 and 2019 (Fig. 1E).

**Fig. 5:**
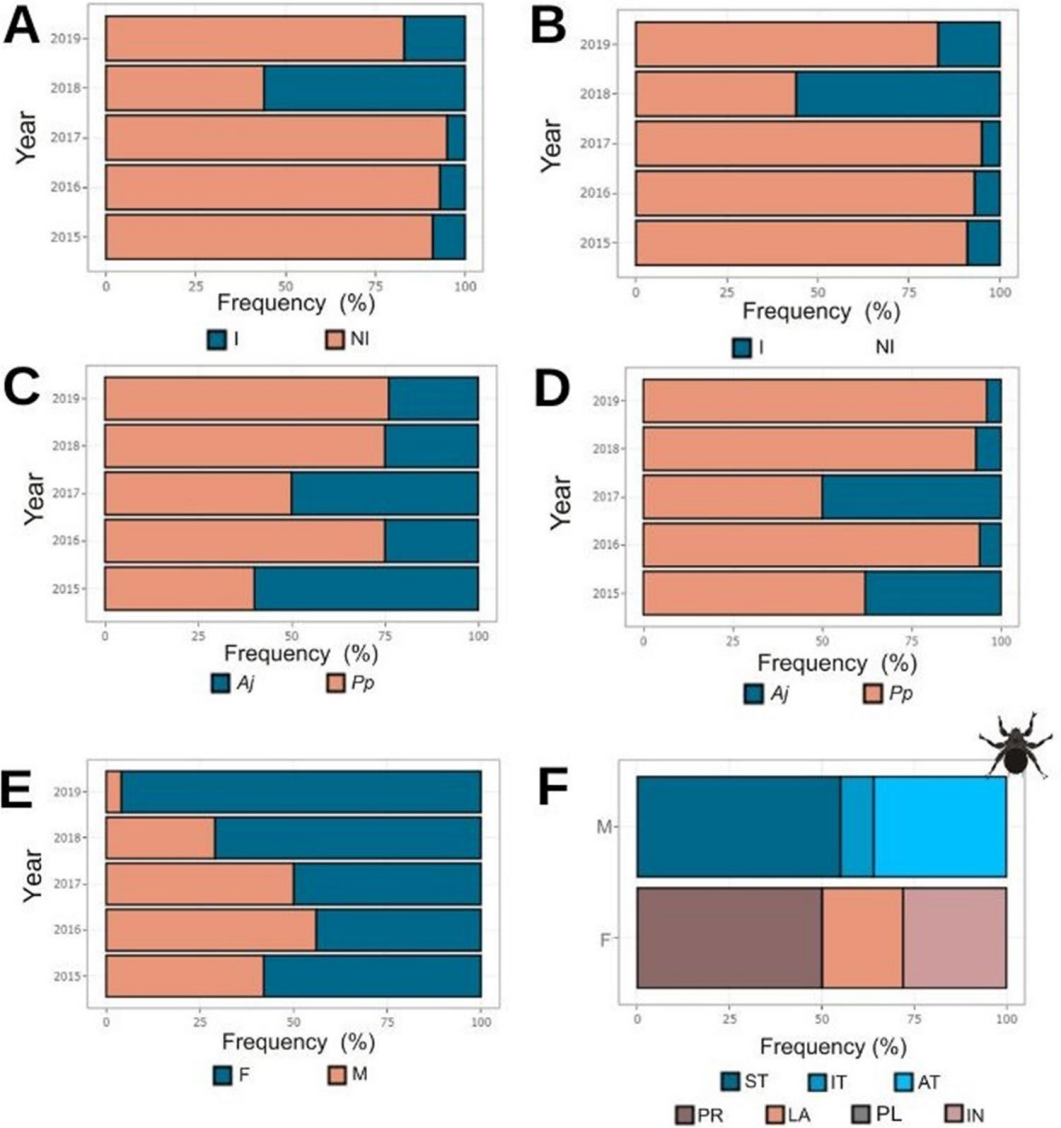
Infestation and abundance of ectoparasites (Streblidae) in two bat species during the dry seasons of the period 2015–2019 in a tropical dry forest of Yucatan, Mexico. Percentage of infested (I) and non-infested (NI) individuals of *Artibeus jamaicensis* -Aj- (**A**) and *Pteronotus parnellii* -Pp- (**B**) over 5 years. Frequency of infected individuals per host species (**C**). Ectoparasite abundance (%) per host species (**D**), host sex (female, F; male, M) (**E**), and host condition (scrotal testes-ST, inguinal testes-IT, abdominal testes-AT, pregnant-PR, lactating-LA, post-lactating PL, inactive-IN) (**F**)

The male condition was not significantly associated with ectoparasite abundance (Table 3); however, on *A. jamaicensis*, we observed that 100% of infested males were reproductive, while on *P. parnellii*, 75% were sexually inactive. In contrast, the pregnant female condition did significantly affect fly abundance (Fig. 5F, Table 4).

**Table 4:** Summary of ectoparasite abundance analysis results for independent variables according to host sex

Component	Estimate	Std. Error	*z* value	Pr( >|z|)
A)				
(Intercept)	− 160.43	1319.94	− 0.122	0.90
AT	14.50	1275.75	0.011	0.99
ST	13.21	1275.75	0.01	0.99
IT	12.67	1275.75	0.01	0.99
Year	0.07	0.16	0.429	0.66
Species (*P. parnellii*)	1.03	0.50	2.04	0.04**
B)				
(Intercept)	− 941.66	164.03	− 5.741	9.43E − 09***
PR	0.52	0.23	2.219	0.02**
PL	− 11.53	1198.39	− 0.01	0.99
L	0.26	0.20	1.331	0.18
Year	0.46	0.08	5.72	1.07E − 08***
Species (*P. parnellii*)	4.71	1.00	4.69	2.73E − 06***

Results based only on male **(A)** and female **(B)** hosts. Male: abdominal testes (AT), scrotal testes (ST), inguinal testes (IT). Female: pregnant (PR), lactating (L), post-lactating (PL). Significant *p*-values are in bold font (0.05**, 0.001***)

## Discussion

In this study, we contribute to the knowledge about Streblidae infra-communities and the effect of host attributes and prevailing environmental conditions in a tropical dry forest during the dry seasons over 5 years on bat fly load; specifically, the infestation and abundance of bat flies in the Jamaican fruit-eating bat *A. jamaicensis* and the common mustached bat *P. parnellii*.

### Description of parasite load in the host community

Our general description of ectoparasite load in bats from the KKBR over four consecutive dry season periods reveals low richness and prevalence but high aggregation. Except for *N. coxata*, the prevalence and mean intensity values recorded in this study are lower than those previously registered in bat species of several localities of Yucatan (Cuxim-Koyoc et al. 2015), and even the values reported during the dry season of other regions of Mexico where TDF is also dominant (Salinas-Ramos et al. 2018). The high aggregation (D) values suggest that most bat fly species in the dry season parasitize few hosts. Previous studies show that high aggregation values translate to most ectoparasites occurring together rather than individually (Barbier and Graciolli 2016; Barbier et al. 2019), and there is evidence to indicate that host attributes, home range, root size and complexity, immunocompetence, and social structure all influence parasite aggregation (Presley 2011).

Our records of bat ectoparasites in this undisturbed TDF represent 27% of the species richness recorded for Streblidae in Yucatan. Three species (*N. parnellii*, *Trichobius johnsonae*, and *Trichobius sparsus*) are new records from Yucatan, increasing the current species list to 22. These bat fly species were previously recorded in TDF in other regions of the country (Zarazúa-Carbajal et al. 2016; Hernández-Martínez et al. 2019; Salinas-Ramos et al. 2018) and in the same host species (Veracruz-Guerrero and Morales-Malacara 1996; Cuxim-Coyoc et al. 2015; Tlapaya-Romero et al. et al. 2019). The bat flies recorded in this study also represent 27% of the species richness documented in TDF bat communities in Mexico (Zarazúa-Carbajal et al. 2016; Hernández-Martínez et al. 2019; Salinas-Ramos et al. 2018).

Twelve bat species did not harbor bat flies during the 5 years of study, which could be related to their low abundance of those bat species, since at least ten of them presented *n* ≤ 5 individuals, indicating that bat fly infestation is frequency-dependent. On the other hand, the lack of infestation of these bat species could also be influenced by environmental conditions, biology, and roost preferences (Hofstede and Fenton 2005; Bordes et al. 2008). All of the parasitized hosts belonged to the Phyllostomidae and Mormopidae families. Except for *Artibeus phaeotis* (Miller), most host bat species of *n* > 8 were infested. It appears that the frequency of infestation is higher in host species with high abundances (Wenzel et al. 1966; Barbier and Graciolli 2016; Barbier et al. 2019). We recorded high infestation in *A. jamaicensis* and *P. parnellii*. Individuals of the latter species were parasitized by more fly species (*n* = 4), while the rest of the hosts were only parasitized by two species. The latter matches a previous study on a Pacific Island dominated by TDF, which reported *P. parnelli* associated with a high richness of bat flies in both the dry and wet seasons (Salinas-Ramos et al. 2018). Even so, in this study, the richness of bat flies associated with *P. parnelli* in the dry season is lower than that reported by Salinas-Ramos et al. (2018), possibly because the specimens were collected from a colony that shared roosts and where the high abundance allows the co-occurrence of a higher number of bat flies species (see Ter Hofstede and Fenton 2005).

*Trichobius yunkeri* and *N. coxata* are the only two species previously recorded in *P. parnellii* in Yucatan; however, we also detected the presence of *N. parnelli* and *T. intermedius* (*n* = 2) in this bat species. While *N. parnelli* naturally parasitizes *P. parnellii* (Guerrero 1993), *T. indermedius* is considered an accidental species in this case, since it is a primary parasite of *A. jamaicensis* (Peterson and Hurka 1974; Wenzel 1976; Guerrero 1995)*.* In our study area, most individuals of both species of bats were collected from the same cave, which could have acted to facilitate inter-species host transference (Patterson et al. 2007).

### Effect of environmental and host conditions on bat fly infestation and abundance

Our results indicate that the ectoparasite load is higher in *P. parnellii* than in *A. jamaicensis*. Two aspects, not mutually exclusive, could be related to differences in infestation and abundance between the host species. On the one hand, the roosting preferences of each species could explain the differences in bat fly densities. For example, *P. parnellii*, is a cavity-roosting species with records of large colonies in caves (Arita 1993), and this behavior and group size increase the probability of registered higher bat flies densities because the Streblidae pupae in this roost and are transmitted horizontally between the colony (see Ter Hofstede and Fenton 2005). On the contrary, *A. jamaicensis* forms small groups in exposed roost (Arita 1993), conditions that are not favorable for Streblid pupae and that are associated with a low density of ectoparasites (see Ter Hosted and Fenton *Op. cit*). On the other hand, although body condition variables (e.g., weight, size, BBCI) had no significant effect on the infestation parameter, food scarcity during this period would have affected both species. In the tropical dry forest, insect abundance decreases in the dry season (Neves et al. 2014), affecting resource availability for *P. parnellii* (see Salinas-Ramos et al. 2015). In contrast, although it has been shown that the availability of fruits for *A. jamaicensis* decreases in the Yucatan during the dry season (Flores-Martínez et al. 1999), this species is capable of exploiting alternative sources (Heithaus et al. 1975; Fleming and Heithaus 1981; Arita and Martínez del Río 1990). Although speculative, this capability could favor the physiological or immune condition of this species and decrease the levels of infestation or abundance of its ectoparasitic bat flies (see Zahn and Rupp 2004; Knutie 2020). This could be supported by the fact that, in this region, both species of bats inhabit and utilize caves as shelters (Arita and Vargas 1995; Ortega and Arita 1999).

Our prediction of a higher ectoparasite load under severe dry season conditions was only partially supported. Despite the absence of any association (P, MA, MI, D) or relationship (infestation, total abundance) between these parameters and environmental conditions, we observed variations across the years. Other studies conducted in TDF have shown that bat fly prevalence on *A. jamaicensis* and *P. parnellii* does not seem to vary significantly between years when measured in the same seasons (see Tlapaya-Romero et al. 2021). However, although our prevalence values were significantly lower than those reported for these species in other TDF regions due to the associated abundances (Salinas-Ramos et al. 2018, Tlapaya-Romero et al. 2021), we observed that susceptibility to the presence of parasites during the dry season varied from year to year for each bat species. Our results also reveal differences in infestation rates (MA and MI) and the distribution of parasites (D) between years, particularly in *P. parnelli*. We observed a trend similar to that described in the prevalence data, with higher infestation rates in 2018, 2019, and also in 2015. These findings contrast with the results of Tlapaya-Romero et al. (2021), who did not detect differences in these parameters in this bat species between dry seasons, perhaps associated with their study scale (Cave). On the other hand, the ectoparasite aggregation index in 2016 and 2017 showed some consistency between both bat species, with most of them infecting few hosts in those years. Yet, we noted that in *A. jamaicensis*, the streblids remained parasitizing few host individuals most of the time, while in *P. parnelli*, they were evenly distributed along our time scale. Evidence suggests that ectoparasite aggregation patterns are influenced by the behavior-ecology of the host and the parasite species rather than the temporal scale or environment of the host (Presley 2011; Morrill et al. 2022).

We did not find a relationship between infestation (infected/not infected) and the abundance of ectoparasites with any of the environmental variables. For example, we observed high infestation in 2018 for both species, a year characterized by recording the second lowest values of AT, precipitation, and relative humidity. We also detected few records of bat flies for both species in 2016 (7%) and 2017 (2%), years in which the relative humidity presented the highest values, and a high abundance in 2019 (52%), when three of the four environmental variables presented their maximum values (see Fig. 1). Our results support those of Patrício et al. (2016), who found no association between bat fly abundance and environmental variables and physiological host attributes. However, we do not rule out the possibility that climatic conditions at a smaller scale acted to drive differences in abundance. The only study in Mexico that has evaluated the environmental relationship with parasite load indicates that parasite abundance (i.e., mean abundance) in bat species collected in a cave can vary between two different periods of the dry season (see Tlapaya-Romero et al. 2021). At the microclimatic scale, differences in bat fly abundance in *A. jamaicensis* across dry seasons could be associated with temperature variations. However, within the timeframe of our study, temperature (minimum and maximum) did not exhibit significant differences among the years, possibly because our scale of environmental measurement differs, and our variables do not precisely correspond to the scale of roost shelters. This leads us to speculate that the relationship between environment and ectoparasite load depends on the measurement scale and habitat context, such as measurements in roost shelters like caves versus non-enclosed landscapes.

The body condition index (BCI) values of both bat species did not show significant differences between the sexes, and they did not explain the high ectoparasite abundance in females, as reported in other studies (see Tai et al. 2022). The general pattern of the many host-parasite systems shows that males exhibit a high parasite load (Patterson et al. 2008; Pollock et al. 2012). However, the adult females of some neotropical bat species are found in harems, while adult males are solitary. This social structure and dynamic have been previously documented in *A. jamaicensis* in the Yucatan Peninsula (Ortega and Arita 1999) and could explain why the females of both species of bats presented a higher abundance of ectoparasites than the males. Our results agree with the general pattern documented in tropical forest bats with this and other ectoparasite groups (Christe et al. 2007; Patterson et al. 2008). On the other hand, grooming behavior and frequency could explain the abundance of bat flies on female’s condition. The grooming rate in reproductive/lactating females is lower because it is energetically costly (Giorgi et al. 2001) and this could explain why pregnant females harbored higher quantities of bat flies (see Tai et al. 2022).

In conclusion, our study reveals differences in host infestation and ectoparasite abundance among dry seasons that are not associated with any environmental variable and body host condition considered in this study. Our assumption that environmental severity will influence a host-parasite interaction is not conclusive. Despite the fact that the environmental variables did not show a direct effect, it can be observed that the highest values of infestation and abundance of ectoparasites are recorded mainly in years with severe environments. We also report that pregnant females were most likely to be parasitized during the dry season, which generally represents the season of most significant nutritional stress. We consider that studies on the effect of environmental variables on parasite load must be addressed within a microclimatic scale or roosting shelter (see Tlapaya-Romero et al. 2021). Our understanding of bat fly interactions in the biogeographical provinces that concentrate biological diversity in the tropical region is still incipient. Much more study is required in Mexico regarding how environmental variables shape host-parasite interactions. This research is essential if we consider that, under climate change scenarios, some regions with the presence of TDF fragments are threatened due to increased temperatures and reduced precipitation (Prieto-Torres et al. 2016). Determination of how species and their obligate interactions will respond to these processes is challenging. However, the effect may be numerical, functional, microevolutionary, and linked to cascading changes; the net effects of which remain unknown (see Brooks and Hoberg 2007). For example, under these scenarios, euryxenic streblids (those with a broad host range) may respond differently to those that are monoxenic (with high host specificity), perhaps generating the loss or local extinction of interactions that, in turn, could impact the immunological or physiological condition of the host species (see Cumming and Van Vuuren 2006).

### Supplementary Information

Below is the link to the electronic supplementary material.

Supplementary file1 (DOCX 28 KB)
