# Supplementary material for: A Five-Year Study on Infestation and Abundance of Bat Flies (Hippoboscoidea: Streblidae) Under Severe Dry Season Conditions in the Tropical Dry Forest of Yucatan, Mexico
Source: Neotrop Entomol. 2024 Mar 26;53(2):439–54. doi: 10.1007/s13744-024-01130-z (PMC11021260; doi:10.1007/s13744-024-01130-z)
Supplement: Supplementary file 1 — Supplementary file1 (DOCX 28 KB) [file 13744_2024_1130_MOESM1_ESM.docx]

**Supplementary Material**

**Table S1.** Results of multiple comparisons of environmental variables spanning five years (2014-2019) using the Dunn test with Bonferroni correction. Net radiation (A), air temperature (B), relative humidity (C), and precipitation (D). Significant p-values are indicated in bold.

|  | 2015 | 2016 | 2017 | 2018 |
| --- | --- | --- | --- | --- |
| **A)**  2015 |  |  |  |  |
| 2016 | -7.54/ **0.000** |  |  |  |
| 2017 | -3.10/**0.0141** | 5.597/ **0.000** |  |  |
| 2018 | -4.63/ **0.000** | 2.89/**0.02** | -2.24/ 0.18 |  |
| 2019 | -9.18/ **0.0000** | -1.05/ 1 | -7.45/ **0.000** | -4.17/ **0.002** |
| **B)** |  |  |  |  |
| 2015 |  |  |  |  |
| 2016 | 7.89/**0.0000** |  |  |  |
| 2017 | -5.42/**0.0000** | -14.55/**0.0000** |  |  |
| 2018 | 4.11/**0.0003** | -3.76/ **0.0012** | 10.17/**0.0000** |  |
| 2019 | -8.94/**0.000** | -17.49/**0.0000** | -4.60/**0.0000** | -13.39/**0.00** |
| **C)** |  |  |  |  |
| 2015 |  |  |  |  |
| 2016 | -9.79/ **0.0000** |  |  |  |
| 2017 | 10.39/**0.0000** | 21.74/**0.0000** |  |  |
| 2018 | 3.98/**0.0005** | 13.79/**0.0000** | -5.78/ **0.0000** |  |
| 2019 | 1.08/1 | 11.66/**0.0000** | -10.25/ **0.000** | -3.22/**0.01** |
| **D)** |  |  |  |  |
| 2015 |  |  |  |  |
| 2016 | -7.54/**0.0000** |  |  |  |
| 2017 | -3.10/**0.0141** | 5.59/**0.0000** |  |  |
| 2018 | -4.63/ **0.0000** | 2.89/**0.0284** | -2.24/0.1859 |  |
| 2019 | -9.18/**0.0000** | -1.05/1 | -745/**0.0000** | -4.17/**0.002** |

**Table S2.** Results of the correlation analysis between bat-fly load parameters and environmental variables, including Prevalence (P), Mean Abundance (MA), Mean Infestation Intensity (MI), and Aggregation Index (D).

| Specie | Parameter |  | Net radiation (P value) |  | Air temperature  (P value) |  | Relative Humidity  (P value) |  | Precipitation (P value) |
| --- | --- | --- | --- | --- | --- | --- | --- | --- | --- |
| *Pteronotus parnellii* | P |  | -0.50(0.45) |  | -0.70(0.23) |  | 1(0) |  | 0.10(0.95) |
|  |  |  |  |  |  |  |  |  |  |
|  | MA |  | -0.29 (0.40) |  | -0.44(0.99) |  | 0.20(0.56) |  | -0.070(0.83) |
|  |  |  |  |  |  |  |  |  |  |
|  | MI |  | -0.025(0.96) |  | -0.075(0.83) |  | 0.14(0.69) |  | -0.17(0.62) |
|  |  |  |  |  |  |  |  |  |  |
|  | D |  | 0.45(0.18) |  | 0.60(0.06) |  | -0.29(0.41) |  | -0.07(0.83) |
|  |  |  |  |  |  |  |  |  |  |
|  |  |  |  |  |  |  |  |  |  |
| *Artibeus jamaicensis* | P |  | -0.50(0.45) |  | -0.70(0.23) |  | 1(0) |  | 0.10(0.95) |
|  |  |  |  |  |  |  |  |  |  |
|  | MA |  | -0.9(0.083) |  | -0.70(0.23) |  | -0.50(0.45) |  | -0.50(0.45) |
|  |  |  |  |  |  |  |  |  |  |
|  | MI |  | -0.33(0.58) |  | 0.11(0.85) |  | -0.89(0.050) |  | -0.89(0.050) |
|  |  |  |  |  |  |  |  |  |  |
|  | D |  | 0.20(0.78) |  | -0.5(0.45) |  | -0.50(0.45) |  | -0.50(0.45) |
|  |  |  |  |  |  |  |  |  |  |

**Table S3.** Statistical significance and AIC values for each predictor in models considering infestation (A) and abundance values (B) as response variables. AICWt represents the contribution of each predictor to the model. Significant *p*-values are in bold.

| **Infestation** | **Chisq** | **Df** | **Pr(>Chisq**) | **AIC value** | **AICWt** |
| --- | --- | --- | --- | --- | --- |
| Air_TC | 4.47 | 1 | 0.06 | 181.75 | 0.02 |
| NR | 0.11 | 1 | 0.74 | 181.85 | 0.02 |
| Pr | 1.02 | 1 | 0.31 | 182.27 | 0.02 |
| Host BBCI | 0.35 | 1 | 0.56 | 182.96 | 0.01 |
| Host Sex | 0.29 | 1 | 0.59 | 182.96 | 0.01 |
| Year | 120.2 | 3 | **<0.001** | 177.04 | 0.24 |
| Specie | 63.67 | 1 | **<0.001** | 177 | 0.24 |
|  |  |  |  |  |  |
| Abundance |  |  |  |  |  |
| Air_TC | 0.03 | 1 | 0.862 | 181.07 | 0.02 |
| NR | 0.03 | 1 | 0.82 | 182.09 | 0.01 |
| Pr | 0.04 | 1 | 0.84 | 183.75 | 0.01 |
| Host BBCI | 3.28 | 1 | 0.07 | 183.88 | 0.01 |
| Host Sex | 27.40 | 1 | **<0.001** | 173.51 | 0.33 |
| Year | 123.31 | 3 | **<0.001** | 174.58 | 0.25 |
| Specie | 51.302 | 1 | **<0.001** | 174.94 | 0.42 |

**Table S4.** Comparison results based on Fisher's test of prevalence (P) for bat flies collected from A. jamaicensis and P. parnellii over five years during the dry seasons. Significant *p*-values are in bold. Host individual number (N), Male (M) and Female (F).

| Year | 1. *jamaicensis* | | | | *P. parnellii* | | | |
| --- | --- | --- | --- | --- | --- | --- | --- | --- |
|  |  | P (95% CI) | | Fisher’s test |  | P (95% CI) | | Fisher’s test |
|  | N | M | F | (*p*) | N | M | F | (*p*) |
| 2015 | 33 | 11 | 0 | 1 | 6 | 100 | 50 | NA |
| 2016 | 14 | 11 | 0 | 1 | 18 | 13 | 20 | 1 |
| 2017 | 37 | 4 | 7 | 1 | 18 | 13 | 9 | 1 |
| 2018 | 10 | 50 | 50 | 1 | 18 | 64 | 100 | 0.10 |
| 2019 | 30 | 31 | 0 | **0.05** | 21 | 0 | 76 | 1 |
